# Pushing for the same thing on the same set of tracks: a qualitative study exploring the anti-trafficking response in Bihar and Uttar Pradesh

**DOI:** 10.1186/s12889-021-11213-w

**Published:** 2021-06-24

**Authors:** Courtney Julia Burns, Keiko Chen, Hanni Stoklosa

**Affiliations:** 1 University of Michigan Medical School, Ann Arbor, MI USA; 2 University of Hawaii Department of Psychiatry, Honolulu, HI USA; 3 Department of Emergency Medicine, Brigham and Women’s Hospital, 75 Francis Street, Boston, MA 02115 USA; 4 François-Xavier Bagnoud Center for Health and Human Rights, Harvard School of Public Health, Boston, MA USA

**Keywords:** Human trafficking, Forced labor, Sex trafficking, Labor trafficking, Resilience, Community-based

## Abstract

**Background:**

Human trafficking is a critical public health issue particularly pervasive in the Indian states of Bihar and Uttar Pradesh (UP), which share a border with Nepal. Many NGOs are participating in prevention, protection, prosecution, and capacity building initiatives. The aim of this study was to identify factors hindering and enhancing the efficacy of anti-trafficking programs in the region.

**Methods:**

A qualitative study was conducted using semi-structured interviews and focus groups with key stakeholders in Bihar, Uttar Pradesh, and Nepal.

**Results:**

Thematic analysis revealed that the barriers hindering the anti-trafficking movement include police corruption, insufficient enforcement of national law, discrimination toward trafficking victims, inadequate funding, and lack of government involvement. Recommendations for overcoming these barriers were through empowering survivors and increasing cooperation, coordination, and communications between NGOs and the government.

**Conclusions:**

In mitigating these barriers and increasing survivor autonomy, anti-trafficking interventions have the opportunity to create individualized environments for those with an experience of trafficking to thrive. Ultimately, elevating community accountability, honoring individual autonomy, and recognizing the value of the persons with a lived experience of trafficking are critical as we continue to use a public health lens in the fight against human trafficking and for human rights.

**Supplementary Information:**

The online version contains supplementary material available at 10.1186/s12889-021-11213-w.

## Background

Human trafficking is one of the fastest-growing criminal industries in the world and carries devastating public health consequences, with the International Labour Organization estimating that there are 40.3 million victims worldwide [1]. In response to this growing problem, programs of all shapes and sizes have arisen to combat human trafficking, yet data on assisted victims of sex trafficking suggests that 34% were re-trafficked [2]. Many promising practices are currently utilized by nongovernmental organizations (NGOs). A more comprehensive understanding of the context in which their efforts are situated is crucial for their greater success. Specifically, while many NGOs are participating in prevention, protection, prosecution, and capacity building initiatives, their outcomes may be optimized if specific attention is given to the regional nuances of the both the barriers and successes of the anti-trafficking programs. This is particularly true in India, where the regions of Bihar and Uttar Pradesh encompass anti-trafficking programs with the potential to substantially mitigate the trafficking pervasive throughout the area.

### Trafficking in Bihar and Uttar Pradesh

Due to Bihar’s geographical proximity to the international borders of Nepal and Bangladesh, migration due to poverty, and employment opportunities, it is one of India’s most vulnerable states for trafficking. The district of North Bihar is particularly vulnerable because of its open borders to Nepal and the state’s high level of intra- and interdistrict trafficking [3]. Children in the state experience trafficking for reasons including sexual exploitation, adoption, entertainment and sports (for example, acrobatics in circuses, dance troupes, beer bars, camel jockeys), marriage, labor, begging, organ trade, drug peddling and smuggling [3]. Approximately 35,000 to 40,000 children from the state are missing, and according to the Census of 2001, Bihar accounts for 8.9% of the child labor in India in the age group of 5–14 years [3]. Furthermore, a household survey conducted by the Bihar Education Project Council showed that there were 2.3 million out-of-school children in Bihar, from which 0.56 million children were out-of-school due to employment [3].

Uttar Pradesh, with nine districts bordering Nepal, is a source, transit, and destination area for the trafficking of women for commercial sex both within the country and across the border. Commonly, victims are procured from Nepal, transported through Uttar Pradesh, and then sent to Delhi and Mumbai [3]. Further, organized crime rackets in the state kidnap and abduct minor victims, after which the children are reared as their own. Upon adolescence, the victims are prepared for commercial sex, which includes forced employment at dance bars in Mumbai and commercial sex in Middle East countries. This occurs in tandem with the use of oxytocin, an injection that can make adolescent and minor girls look like adult women [3]. Additionally, child labor is also rampant in the Uttar Pradesh carpet belt area and brick kilns industry, with children procured from Bihar for this labor [3]. As per a 2010 National Commission for Protection of Child Rights (NCPCR) report, Uttar Pradesh accounts for the largest share of all child workforce in India, at about 15% [3].

### Existing government anti-trafficking initiatives in India

Given the pervasive nature of trafficking within these Indian states, several anti-trafficking initiatives were implemented by various governmental ministries in the country, in collaboration with NGOs. Understanding the nature of the region’s programs at the time of our study’s data collection is important for contextualizing our findings.

The Ministry of Home Affairs implemented Anti-Human Trafficking Units (AHTU) in a majority of districts across India [3]. The AHTU functioned primarily to coordinate anti-trafficking response among law enforcement, prosecutors, civil society organizations, and NGOs. These anti-trafficking responses included coordinating efforts for criminal prosecution, assisting victims in leaving situations of trafficking, and then caring for them after this occurs. Every AHTU had an appointed nodal NGO that helped law enforcement identify victims and coordinates post-trafficking care [3]. This nodal NGO managed government or private shelters for rehabilitation and the subsequent reintegration or repatriation, if applicable. NGOs additionally served a vital role in helping law enforcement understand the regional nuances of trafficking in their specific area, as they were closely tied with the community [3]. Often, parents contacted the NGOs directly about missing children instead of approaching law enforcement. Then, it was the role of the NGOs to coordinate with law enforcement to investigate and conduct an operation to dissolve situations of trafficking and aid victims, with police conducting the operations under predetermined protocol. The NGOs monitored anti-trafficking responses to ensure follow-through on these cases [3].

The Ministry of Women and Children developed schemes and protocols to target services toward victims of commercial sexual exploitation of children (CSEC). This included the ministry’s development of manuals for various providers, as well as the protocols for best practices before, during, and after assisting people exit trafficking. In 1998, the government formulated a National Plan of Action to Combat Trafficking to reintegrate women and child victims of commercial sexual exploitation into society [4]. Schemes, or anti-trafficking programs, were formed to implement this goal of reintegration, which included the Ujjawala Scheme, Swadhar Scheme, and Integrated Child Protection Scheme.

The Ujjawala Scheme began in 2007 and functioned to assist women and children trafficked for commercial sexual exploitation in exiting trafficking, in addition to their rehabilitation, reintegration, and repatriation. The scheme also coordinated widespread trafficking prevention and awareness efforts at the community level [5]. However, there were no standard operating procedures established, and as such, this scheme’s projects were not monitored to a set standard [3].

The Swadhar Scheme was launched in 2002 for ‘women in difficult circumstances’ as a continuation of the ‘Short Stay Home’ scheme, which provided temporary accommodations and rehabilitation services for homeless women and girls [6]. Food, shelter, clothing, counseling, vocational training, medical care, and legal aid were provided, and Bihar had three shelters under this scheme. While the Ujjawala Scheme served as the primary scheme for trafficked victims, it did not exist in all cities or districts and its resources were finite. Therefore, in some cities or districts, the Swadhar scheme was a safety net for those victims whose needs were not met under Ujjawala [6].

In 2009, the Integrated Child Protection (ICP) Scheme was launched and provided funding for most states, who then dispersed these funds to the state and NGO-operated components. This led to efforts such as the 24-h CHILDLINE hotline, which functiond to connect CSEC victims to services in their area ranging from shelters to medical aid. It handled tens of thousands of calls per year from across the country and had a well-designed documentation system to identify types of services to which the children were linked [3].

The Ministry of Labour and Employment established the Protocol on Prevention, Rescue, Repatriation, and Rehabilitation of Trafficked and Migrant Child Labor. The ministry also assembled a “rehabilitation package” for bonded laborers to receive INR 10,000 from federal funds in addition to a separate state-funded package. This state package included allotment for a house-site, agricultural land, farm animals, skill development training, and education for their children. However, no evaluation has been conducted on the actual success in implementation this rehabilitation package or its impact [3].

Lastly, the Initiatives on Missing Children Department has been involved in anti-trafficking efforts since 2004, when the National Human Rights Commission Action Research Report highlighted the link between missing persons and human trafficking in India. It was not uncommon in the Bihar-Uttar Pradesh region for significant numbers of missing children to be left uninvestigated [3].

### Aims

This study aimed to identify the factors hindering and improving the efficacy of anti-trafficking programs in the Indian states of Bihar and Uttar Pradesh along the India-Nepal border.

## Methods

A qualitative study was conducted using semi-structured interviews and focus groups with key stakeholders in Bihar, Uttar Pradesh, and Nepal. Qualitative methodology was chosen given the exploratory nature of our aim. The study received ethical approval from the institutional review board of the Harvard T.H. Chan School of Public Health and from the institutional ethics committee of the Society for Integrated Development and Reconstruction in India. We gathered key stakeholder input through semi-structured interviews and focus groups.

### Research team and reflexivity

Interviews were conducted by HS, a female MD, MPH who has conducted multiple published qualitative studies in the past. No relationship with the participants was established prior to the study commencement. The participants were aware that the researcher was conducting the research to improve the anti-trafficking response in the region, given the fact that almost no peer reviewed literature has come out of the region. The interviewer’s bias that the anti-trafficking response could be improved was stated.

### Study design

This study took a content analysis approach. Participants were selected via snowball sampling until saturation was achieved [7]. Participants were approached initially via email. No participants were younger than the age of 16. A total of 43 individual and 7 focus group interviews were conducted in June 2014. No participants refused to participate. The interviews took place in a private location of the interviewee’s choosing. In some cases, due to space constraints, other staff may have been in earshot of the interview, but the utmost care was taken to find the most private setting possible. The key stakeholder interviewees fell into the following categories: NGO employees who implement anti-trafficking programs, anti-trafficking program participants, government officials, law enforcement, and prosecutors.

The semi-structured interview guide which was developed for this study had two main areas of inquiry on 1.) identification of problems hindering the progress of the anti-trafficking movement and 2.) recommendations for overcoming these issues to accomplish the common goal of helping people exit the cycle of trafficking. No repeat interviews were carried out. The interviewer provided assurances of anonymity, answered questions regarding participation, and obtained informed consent immediately before each interview. Interviews were audio recorded then transcribed and translated for data analysis. Field notes were made during and/or after the interview. Interviews lasted between 40 and 90 min. Each transcript was reviewed for accuracy against the recording and coded using NVivo software. Due to logistical constraints, transcripts were unable to be returned to participants for comment or correction. A unique identifier linked participant interviews and survey data. All identifying information was removed from transcripts, including the interviewee’s organizational affiliation, to protect anonymity.

### Analysis

Using an inductive content analysis approach, a coding structure was developed [8]. The transcripts were then independently coded by two separate researchers, KC and TK, compared for agreement, and finalized. Participants did not provide feedback on the findings due to logistic constraints. The coding tree with themes and subthemes can be found in Additional file 1. Findings are presented in the results section.

## Results

### Barriers to anti-trafficking initiatives

Thematic analysis revealed that several common problems were hindering the anti-trafficking movement that victims and leaders alike believed needed to be addressed. These barriers include police corruption and insufficient enforcement of national law, discrimination toward trafficking victims, inadequate funding, and lack of government involvement. Below we summarize interview content, including quotes from interviewees.

### Police corruption and lack of enforcement of national law

Interviewees reported that despite the existence of trafficking laws on the national level, the implementation and enforcement of these laws had been neglected, often hindered by limitations of capacity and resources. This fragmentation of enforcement of the national law was problematic. Interviewees stated that national law has a very high standard, and often there were limited cooperation from the Child Welfare Committee (CWC) and the police to appropriately comply with it, largely due to the governmental staff’s lack of awareness towards the law and its application. There were cases in which procedural issues to document and/or declare bonded labor resulted in opportunities for police corruption. Examples of corruption mentioned by respondents included utilizing court date delays to tamper with evidence, falsifying evidence, relying on the unscientific age verification test, incorrectly applying the wrong legal section (e.g. rape cases are not filed as rape), tampering/threatening the witness, bribing officials, and rejecting bail.

Furthermore, respondents mentioned that there had been several instances of police officers forcing trafficked girls to change their statements against their traffickers and tampering with medical records and age verification. This allowed for a more lenient sentence for traffickers, delay of investigations, and the prolongment of the time trafficked girls were kept in the police station.

#### Criminalization of victims

As a result, trafficking survivors were sometimes criminalized instead of their traffickers, rather than being treated as victims in need of assistance and protection. An interviewee from an NGO narrated one instance in which innocent individuals were taken away by the police, some of them previous victims of trafficking.

“There was one incident where a girl named Varuna [pseudonym] disappeared. When the police came to investigate, my brother was tied with a chain and dragged away by the police. Other women were also taken under the premise that they were ‘bought.’ Some of these girls had already been rescued from the flesh trade. Now the same exploitation is happening to them.”

These instances pointed to the pervasive nature of police corruption throughout the system. While several NGOs have filed public interest litigations against the police, they expressed frustrations with the difficulty in properly filing cases and getting such cases tried in court. Even when cases proceeded to court, the NGOs continued to encounter corruption and tampering of evidence.

#### Distrust of police

As a result of the police officers’ corrupt acts, victims felt extremely uneasy in the presence of the police. One survivor stated that the police did not respect their privacy nor listen to their grievances:

“The police do not let us live in peace. We can’t sleep, in fear of the police, because they might come any time for interrogations. They ask us to open our wardrobes, suitcases, and then our personal belongings. They never listen to our grievances.”

### Discrimination

Not only have trafficking victims experienced mistreatment by the police, but they have also faced widespread discrimination by others in the community, one example being their mistreatment by merchants. One survivor stated that when merchants learned that they are trafficking victims, they significantly raised the prices:

“Whenever we go shopping, if the shopkeepers know we are trafficking victims, they automatically increase the price of goods. Taxi drivers also ask us for more money. It is not just the police, everyone harasses us here.”

### Lack of funding for NGO activities

Many NGOs have expressed limitations in their activities due to a lack of funding and resources. One grassroots NGO said that working in anti-trafficking is difficult and that one NGO alone cannot make a difference against the most organized crime in the world today, especially as operations to help people exit the situations of trafficking are not always followed by a criminal case. Multiple respondents discussed how it was clear that an NGO required funding to survive in this industry, but fundraising required time and manpower, which detracted from fieldwork. This challenge was immense for them, as they believed that grassroots NGOs understand the issue of trafficking and what was happening on the ground and were a significant source of support in villages.

#### Lack of investment in infrastructure

Furthermore, interviewees reported that finding placement for trafficking victims after leaving their situations of exploitation was extremely challenging due to the lack of adequate safe havens. This created potentially retraumatizing environments for victims that had exited trafficking as they may have also experienced prolonged stays in the police department due to the presence of inadequate and inappropriate shelter homes. One NGO staff person remarked,

“There lacks infrastructure for trafficking victims, especially the mentally challenged. They push all the mentally challenged women to this particular shelter home, which is supposed to house vulnerable people who are in need of care and protection. But these mentally challenged victims need more psychological support than the average person and there isn’t adequate infrastructure that gives them this support.”

### Lack of government involvement

Some of the government’s role was to provide shelter for children, documentation (e.g. release certificates, provisions, and sustainable rehabilitation solutions), and home verifications; however, many trafficking victims expressed a lack of governmental support and action, and the need for more collaboration between NGOs and the government. One government official stated,

“Two years ago, the government was not thinking so much about this type of issue. It is the NGO who actually took charge of setting up a connection with the government and only then do villages start functioning in the districts.”

Moreover, while it is the role of the government’s CWC to handle trafficking cases in terms of home verification, assistance for children in exiting situations of trafficking, parental identification, and linkage to shelters, the number of trafficking cases far surpassed the government’s capacity to handle them. The bureaucracy, lack of efficiency, and politically influenced CWC team member selection further hindered the prosecution of trafficking cases. In turn, these inefficiencies delayed the release of children from the shelter. In addition, CWC only worked during business hours, which posed a challenge when anti-trafficking operations were performed outside of these times.

#### Low capacity

Additionally, there were delays in aiding people in exiting situations of trafficking due to low capacity of the government officials, who must follow all protocols, and accordingly could not always act quickly enough. This lead to some children being trafficked away prior to anti-trafficking intervention at the site of concern. Low capacity also created a large backlog of cases, which was exacerbated by high turnover of staff, including investigation officers being transferred at any time.

### Interviewee recommendations

During our research, community members shared their thoughts regarding the problems that hindered the progress of the anti-trafficking movement and two major themes emerged. The first was to increase cooperation, coordination, and communication between the government and NGOs. The second was to empower trafficking survivors. Interestingly, interviewees discussed empowerment as the main tool of reform above any other recommendations. They believed empowering trafficking victims by giving them the tools needed to rebuild their lives post-trafficking was the most sustainable solution to prevent them from falling victim to trafficking again.

### Increase cooperation, coordination, and communication

Respondents discussed how heightened collaboration between the government and NGOs would sustain a robust, systematic approach to anti-trafficking programs’ shared goal of helping people permanently exit the cycle of trafficking. While there were many government schemes, programs, laws, and legislations in place to address trafficking, the efficacy of these anti-trafficking measures in accomplishing the programs’ common goal was hindered by government corruption. Subsequently, it then became the NGO’s responsibility to investigate this and ensure that protocols were being followed. In doing so, NGOs could ensure that trafficking victims successfully gain access to government schemes, programs, and legal compensation by following standardized operating procedures and protocols in line with existing government and law enforcement efforts. If existing processes established by the government and law are enhanced and executed properly by NGOs, then victims are more likely to successfully remain removed from trafficked situations. For example, the government-run CWC’s scope encompassed the removal of victims from exploitation as well as monitoring. In that realm, there is significant room for collaboration with NGOs to provide necessary treatment for victims. However, one interviewee from an NGO stressed that NGOs currently must take it upon themselves to actively facilitate better communication with the government:

“We need more communication with the government, but the government has to come in front and take the initiative. It is important that we have this communication established because if we are not working together, only parallel to each other, then there will not be as much momentum as if we were pushing for the same thing on the same set of tracks.”

Another interviewee from an NGO expressed similar sentiments, stressing also that active efforts on the part of the government to reach out to and work with NGOs would be greatly appreciated:

“The government can improve NGO-government relationships if they take the time to call us, listen to us, or share news and updates with us.”

Furthermore, a common sentiment expressed by NGOs was that they felt a lack of adequate infrastructure provided by the government. In one interview, a member of the CWC said that there is a need for higher quality facilities to better serve every member of the CWC as they pursue their full potential.

“I think the government should improve the facilities given to the CWC, as it is currently not good enough now.”

While interviewees touched upon several issues between the government and NGOs, they also mentioned where they believe there have been existing successes. These successes demonstrated the immense anti-trafficking potential inherent in increased cooperation, coordination, and communication between the government and NGOs. Notably, interviewees believed that one very important success by the government is recognition of the contributions NGOs make to the anti-trafficking movement in India. According to one NGO employee,

“The government acknowledges that NGOs are doing marvelous work. They do not deny the number of cases that have been entrusted on us and the successes we have had with helping trafficking victims get back on their feet.”

Interviewees believed that this governmental recognition is responsible for many of the NGOs’ ability to make positive impacts on trafficked victims through conducting successful operations to dissolve situations of trafficking, providing safe shelters and centers for children, providing education, providing life skills training (e.g. computers, sewing) and vocational training, and giving children a future. Governmental recognition of NGOs’ abilities is also important for turning research into policy. An NGO employee stated,

“The research and studies that NGOs have put before the government allow the government to establish shelter homes, rescue teams, provide more compensation for the victims. The data and figures that we collect promote change and put pressure on policymakers and bring awareness to the public.”

Ultimately, if steps are taken to address interviewees’ concerns and build upon these current successes to increase dialogue, collaboration, and notions of shared responsibility between the government and NGOs, the needs of trafficking victims will be better served as they pursue safety, healing, and community reintegration.

### Empowerment of trafficking victims

Interviewees representing NGOs and trafficking victims alike believe in the efficacy of community centers, CWCs, community vigilance committees, and microsavings groups in preventing cases of re-trafficking. They also recognized the harmful impacts of discrimination and lack of acceptance from society on victims. Many interviewees expressed the need to sensitize communities, police, and government workers to promote awareness of trafficking, and decrease this stigma. Increased awareness also has the potential to inspire vigilance within the community and cause the recognition of traffickers and illegal activity. Furthermore, many felt that it was important to raise awareness within vulnerable populations about the lure of foreign employment as a front for trafficking. Members of this community were often unaware of the various forms of trafficking, as well as the legal system and their rights. The following include four empowering avenues of increasing anti-trafficking efficacy mentioned by interviewees.

#### Community centers

One interviewee started as a volunteer at one of the community centers and is now a program manager. They discussed projects that brought members of both the trafficking and local community together in a joint effort to decrease the risk of re-trafficking.

“With the help of local people who want to break the lock that threatens to chain their children to the trafficking business, we have built a large community center. The girls that have been saved from or are trying to escape trafficking work for the community in these centers. This is a sign of our success.”

#### CWCs

Members of the CWC stressed that the collective pressure of a committee to demand justice from the system is not only a sign of the strength of an organization, but also a convincing reason to invest in and join CWCs.

“When the local police took away our innocent girls, we went to the Inspector General of Police who listened to us and made a phone call to get the girls released. At midnight, they were dropped off in their houses. This was an organizational achievement and showed the strength of persistence and organization.”

“The girls here have lots of talents, like singing, dancing, painting, but they score very low in exams because the teachers who know they have been trafficked before deliberately give them low marks. The committees help by talking to the teachers and demanding just treatment for the girls.”

Members also stressed that the committees educate and give children skillsets that help them secure a better future.

“The committees run educational classes that give the children confidence. They are learning skills and progressing with their lives.”

“The committee is full of benefits. Our children were previously uneducated, as we could not afford to send our children to school. Our kids now know how to read and write. They taught our kids very well.”

#### Community vigilance committees

Interviewees highlighted the role of women in community vigilance committees and the overall importance of women’s empowerment in the anti-trafficking movement. Empowering women both economically and politically through self-help groups and community vigilance groups created financial independence and promoted participation in local governments, ultimately fostering intergenerational resilience and prosperity.

“With the help of local people and leaders, we form community vigilance groups. These groups give women political empowerment to address and raise trafficking issues to the local administration.”

“Women in the community vigilance committees sell bangles and sell them from village to village. At each village they collect information. If any suspected cases of trafficking arise, they inform the community vigilance leaders.”

#### Microsavings groups

Many agreed that the first important step toward the prevention of re-trafficking is to understand the origins of trafficking. Interviewees agreed that even if victims exit trafficking and are reintegrated back to their families, they will be re-trafficked if the original situation that they came from does not change. Such risk factors identified were poverty, socioeconomic factors, family issues, cultural norms, lack of education, debt bondage systems, and gender discrimination.

Poverty was the fundamental driving force for trafficking and re-trafficking and was what shaped socioeconomic disparities and family dynamics. One NGO said that the lack of livelihood options in villages created a power imbalance between traffickers and victims, and that it was important to provide livelihood opportunities to stop trafficking. Victims are lured by the traffickers’ money and the false prospect of a better life, causing all potential victims experiencing financial vulnerability to face an increased risk of trafficking and re-trafficking. For this same reason of financial insecurity, many children were pushed into trafficking by their families. Families believed that “if I send my child, I can earn more money”, and saw their children as the breadwinners of the family. One NGO member described the state of the community that they worked in: “they send their children to earn. To earn, to survive their livelihood”, as households often included eight to 12 children each, rendering it difficult to take care of them all. Because families’ financial constraints often pushed them to send their children into trafficking, interviewees recommended that starting microsavings groups would help to break the chain of re-trafficking and bonded labor. Microsavings groups are informal, community-based savings groups utilized globally as a method of fostering community resilience and financial stability. One committee member stated:

“We found that if we could organize the women, give them the opportunity to save a little money with some group members, it makes a big difference. Last year, these women received a bank loan because they were able to deposit regularly at the bank. Because a lot of these women are illiterate, it is important that we teach them how to keep accounts, how to manage the savings groups, how to authorize meetings, etc.”

Another committee member stated:

“Savings groups can be used as a prevention method for keeping women out of trafficking situations. Economic development activities like income generation in local trades help women earn money and develop economic self-sufficiency so that they will not willingly put themselves into the trafficking business.”

Core to these recommendations from respondents is that financial resilience is necessary at the individual and family level to prevent someone from being re-trafficked.

## Discussion

This study points to the critical need for enhancing coordination between NGOs and the government, decreasing corruption, and empowering victims. Such approaches are core to a public health response to trafficking, as they are necessary for prevention of both trafficking and re-trafficking [9]. These focused efforts have the potential to improve the anti-trafficking response along the India-Nepal border, and examples and specific practices for doing so can be drawn from the literature.

Nonprofits and the government exist in a web of mutuality which is not unique to the anti-trafficking response. There are a variety of mechanisms that have worked in other contexts to increase coordination between NGOs and the government. Core tenets of improving this coordination include “the standardization of government applications and reporting requirements, formal feedback mechanisms for evaluation of organizational activities, development of NGOs’ organizational capacity to apply for and implement government grants and contracts, and increased public education and awareness regarding the importance of government grants” [10].

Mirroring these international standards, the government of the Netherlands has provided an outline of best practices for interprofessional collaboration specifically in the anti-trafficking movement, with its contents highly applicable to the joint work of government and NGO stakeholders in Bihar and Uttar Pradesh. It encourages the use of protocols to clearly delegate responsibilities to be handled by each organization, especially in the case of operations performed to curtail situations of trafficking. Examples of important considerations include deciding which organization is responsible for funding victim assistance and for how long, confidentiality rules between organizations, and the creation of an evaluation mechanism to identify needed areas of growth in the NGO-government partnership [11]. In states such as Bihar and Uttar Pradesh, given the large number of NGOs and anti-trafficking programs responding to a particularly high level of trafficking, following best practices such as these may lead to increased anti-trafficking success.

The pervasive nature of corruption and its devastating impacts on anti-trafficking efforts described in this study have been the focus of prior India-specific and global trafficking literature [12–14]. However, evidence is limited, as there are practical and political challenges in collecting data on the impact of corruption on anti-trafficking efforts. In addition to more data, concerted political commitment to target resources toward tackling corruption and the development of good governance supported by infrastructure and transparency are necessary [14].

Across the anti-trafficking literature, co-production of interventions and survivor-led interventions are critical for empowering survivors. Laurie et al. describe a Nepalese program functioning as a marriage between victim empowerment and increased interprofessional collaboration - an NGO operated by formerly trafficked women that carries a focus on cultivating external partnerships [15]. Another successful organization with similar empowerment of trafficking survivors can be found in the United States in California. Thus far, it has drawn upon the expertise and skills of the survivor-staff members to conduct anti-trafficking trainings for over 10,000 professionals encompassing a variety of fields, including those within government organizations [16].

NGOs should make a concerted effort toward integrating trafficking survivors as equal partners in the anti-trafficking movement. The Women’s Consortium of Nigeria (WOCON), Children in Solidarity with Africa and the World (ESAM) in Benin, and the Association for the Advancement and Defense of the Rights of Malian Women (APDF) recognize the importance of using community-based interventions for anti-trafficking work [17]. They place a particular emphasis on reflecting survivor opinions in policy agendas, with WOCON saying, “the strength of [projects] is in the active involvement of the community dwellers in identifying the causes and finding solutions to the prevention of trafficking” [17]. Engaging trafficking survivors in an activist role not only provides stable employment opportunities, but also cultivates their leadership experience and familiarity with anti-trafficking resources. Importantly, it also provides a natural channel for amplifying to the government and other stakeholders the true, rather than perceived, needs of victims.

These anti-trafficking programs and guidelines highlight that successful advocacy is best born from a co-production of knowledge that spans across professional affiliations and is dependent on a network of trust built among all involved - in this case, NGOs, the government, and the trafficked persons they serve. This trust underpinning anti-trafficking efforts’ efficacy is critical and can lead to long-term strategic network building and the implementation of activist-informed policy optimally effective for helping victims permanently exit the cycle of trafficking. The magnitude of trust serving as a determinant of program success was phrased eloquently by authors profiling the survivor-led NGO in Nepal, who said trust is, “at the heart of a politically engaged understanding of collaboration which aims to raise the profile and listen to the voices of excluded marginalized actors” [15]. If this lens of empowerment-inspiring trust was seen in Bihar and Uttar Pradesh, significant and long-lasting change toward health and prosperity may be observed in the lives of thousands of individuals experiencing trafficking in these Indian states annually. To bring this goal of empowerment to fruition, it is also vital that NGOs intervening in trafficking in India place greater importance on understanding the lived realities and needs of victims, rather than externally imposing their ideas of what victims may find essential [18].

## Conclusions

From a public health lens, in addition to a focus on prevention of people being re-trafficked, we also recommend an intentional shift away from ‘rescue’ being the yardstick for considering anti-trafficking efforts successful. Success must instead be defined through survivor-driven outcomes, with a focus on practices designed for empowerment and autonomy. By reconceptualizing anti-trafficking efforts’ efficacy in this way - providing less emphasis on ‘raid and rescue’ operations and more emphasis on addressing the structural underpinnings of each persons’ pull toward trafficking - we have the opportunity to create environments for victims to thrive. This also contributes to building communities more deeply valuing the lives of one another. Ultimately, elevating community accountability, honoring individual autonomy, and recognizing the value of the persons with which we each interact are critical as we continue to use a public health lens in the fight against human trafficking.

## Supplementary Information

**Additional file 1.** Improving the anti-trafficking response in Bihar and Uttar Pradesh, India: Coding tree of key themes and subthemes.

## Data Availability

Deidentified data may be made available upon request to the corresponding author.

## Abbreviations

AHTU: Anti-Human Trafficking Units
APDF: Association for the Advancement of the Rights of Malian Women
CSEC: Commercial Sexual Exploitation of Children
CWC: Child Welfare Committee
ESAM: Children in Solidarity with Africa and the World
ICP: Integrated Child Protection (Scheme)
INR: Indian Rupee
NCPCR: National Commission for Protection of Child Rights
NGO: Nongovernmental organization
WOCON: Women’s Consortium of Nigeria
