# Supplementary figures and images for: Pushing for the same thing on the same set of tracks: a qualitative study exploring the anti-trafficking response in Bihar and Uttar Pradesh
Source: BMC Public Health. 2021 Jun 24;21:1204. doi: 10.1186/s12889-021-11213-w (PMC8223280; doi:10.1186/s12889-021-11213-w)

**Appendix A**

Coding tree depicting key themes and subthemes


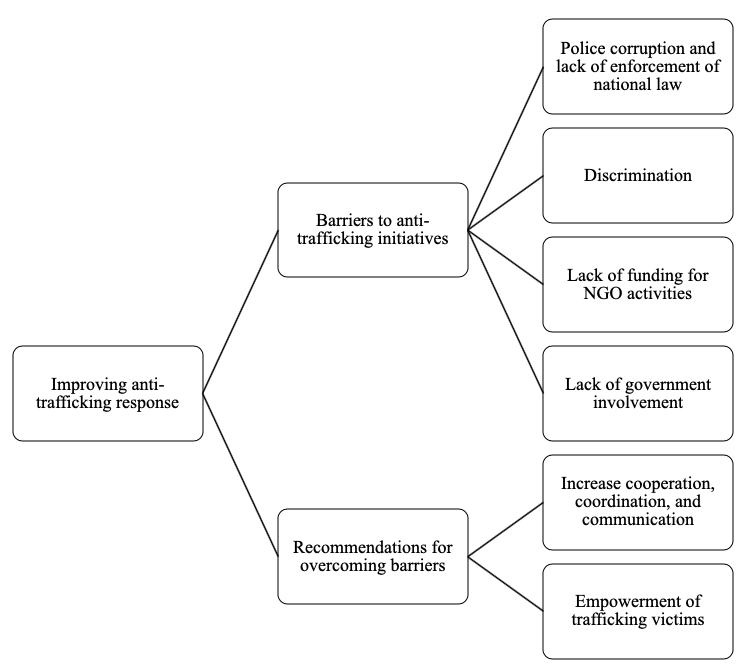

Supplement: Supplementary file 1 — Additional file 1. Improving the anti-trafficking response in Bihar and Uttar Pradesh, India: Coding tree of key themes and subthemes. [file 12889_2021_11213_MOESM1_ESM.docx]
